# Activity Engagement Across Stages of Cognitive Health

**DOI:** 10.3390/healthcare13212712

**Published:** 2025-10-27

**Authors:** Cynthia Williams, Corinne Labyak, Andrea Arikawa, Anna Watermann, Wanyun Chou, Adewale James, Peter Holland, Mariana Dangiolo, Michal M. Masternak, Adam Golden, Shalini Jain, Hariom Yadav

**Affiliations:** 1 School of Global Health Management and Informatics, University of Central Florida, Orlando, FL 32816, USA; wanyun.chou@ucf.edu; 2 Brooks College of Health, University of North Florida, Jacksonville, FL 32224, USA; c.labyak@unf.edu (C.L.); a.arikawa@unf.edu (A.A.); n01222666@unf.edu (A.W.); 3 USF Centre for Microbiome Research, University of South Florida, Tampa, FL 33612, USA; adewalesegunjames@usf.edu (A.J.); jains10@usf.edu (S.J.); hyadav@usf.edu (H.Y.); 4 Department of Neuroscience, I-Health, Schmidt College of Medicine, Florida Atlantic University, Boca Raton, FL 33431, USA; holland@health.fau.edu; 5 Orlando Veterans Affairs Healthcare System, Orlando, FL 32827, USA; mariana.dangiolo@ucf.edu (M.D.); adam.golden@va.gov (A.G.); 6 Department of Internal Medicine, University of Central Florida College of Medicine, Orlando, FL 32827, USA; 7 Burnett School of Biomedical Sciences, University of Central Florida College of Medicine, Orlando, FL 32827, USA; michal.masternak@ucf.edu; 8 Department of Head and Neck Surgery, Poznan University of Medical Sciences, 61-701 Poznan, Poland

**Keywords:** aging, social engagement, cognition, dementia, meaningful activities

## Abstract

**Objective**: This study aims to examine activity engagement across stages of cognitive health among older adults. **Methods**: We used a cross-sectional study analysis of baseline data collected as part of the prospective Microbiome in Aging of Gut and Brain (MiaGB) longitudinal study; the study period was August 2022 to December 2023. Health history and activity engagement questionnaires and the Montreal Cognitive Assessment (MoCA) were used to examine the study objective. One-way ANOVA and chi-squared tests, with Bonferroni post hoc analyses, assessed group differences. **Results**: The weighted samples reflected 417 participants: 54% females, 70.7% White, with an average age of 72 (±8.7) years, 90% with at least high school education, and 75% self-reported medium income status. Results suggested that individuals who scored ≤17 points on the MoCA had an average age of 84 years, were White, non-Hispanic, female, had less than a high school education, and medium income status (*p* < 0.001). Significant differences were found in active engagement in all health behaviors (*p* < 0.05). The frequency of engagement in activities was all statistically significant (*p* < 0.05), except the frequency of looking after grandchildren (*p* > 0.05). Older adults who scored ≤17 MoCA points reported higher rates of hypertension, osteoarthritis, and depression compared with individuals who scored higher on the MoCA assessment. **Conclusions**: Older adults with lower cognitive status report a higher rate of clinical ailments and have less engagement in meaningful activities. We should promote meaningful activities to improve the quality of life in older adults with decreased cognition. We make recommendations for appropriate modifications for activity engagement across cognitive health levels.

## 1. Introduction

As the population ages, Alzheimer’s Disease and Related Dementia (ADRD) is increasingly a public health challenge. Globally, the incidence of ADRD is expected to be approximately 150 million cases in 2050 [1]. The number of ADRD cases in the United States (US) is projected to increase from 6 million in 2023 to an estimated 14 million by 2060 [2]. With the lack of curative or pharmacotherapy treatment and the growing prevalence of ADRD, we must seek ways to maximize the quality of life through meaningful activities for older adults who experience cognitive decline.

Meaningful activities are described as actions that increase positive emotions, improve quality of life, and support performance in activities of daily living [3]. Allison and colleagues (2022) defined meaningful activities as enjoyable actions related to personal interest and values [4]. Despite cognitive challenges, engaging in activities allows older adults with ADRD to participate in purposeful communication, demonstrate agency, and receive support [5]. It also provides social connectedness, physical activity, and mental stimulation [6]. Social and physical engagement in meaningful activities may reduce the progression of cognitive decline [7,8]. Despite these benefits, a study suggested that engagement in meaningful activities and social relationships decreased after a diagnosis of ADRD, indicating a positive association between cognitive health and engagement in meaningful activities [9,10]. In addition, many older adults with ADRD were in passive environments that fostered physical inactivity and promoted agitation [11]. To promote quality of life, we must encourage meaningful engagement among older adults across a range of cognitive levels. Older adults with decreased cognitive function can participate in walking and structured exercise programs, as well as activities such as gardening, music, and visual arts [12]. Additionally, Huizenga and colleagues (2023) found that people with dementia could engage in routine household activities, social engagements, and shared experiences with loved ones [12]. Studies indicated that older adults with mild dementia reported that their participation in physical, recreational, social, cultural, and spiritual activities would likely decrease in the future [13]. The decline in engagement was attributed to reduced physical mobility, caregiver burden, or the absence of a caregiver [12]. Older adults with increased agitation and a lack of coping strategies may also deter engagement in meaningful activities, particularly when places or people are unfamiliar or cognitively demanding [13]. The proposed activities should consider the whole person, encompassing both physical and mental capabilities. To support quality of life, it is imperative that we consider health behaviors and clinical diagnoses that are often analyzed separately. This study aims to describe activity engagement at various levels of cognitive status. Additionally, we examine demographic and clinical factors that warrant consideration alongside cognitive status and activities. We hypothesize that meaningful engagement declines as performance on the cognitive assessment, Montreal Cognitive Assessment (MoCA), declines.

## 2. Materials and Methods

### 2.1. Study Design

This study represents a cross-sectional study analysis of baseline data collected as part of the prospective Microbiome in Aging of Gut and Brain (MiaGB) longitudinal study. The MiaGB Consortium is a multisite, interdisciplinary collaboration among several academic institutions in Florida. The Consortium aims to understand how microbiomes influence our gut, brain, and muscle health during the aging process. This study reflects the initial study recruitment period, August 2022 to December 2023. The University’s Institutional Review Board approved the study protocol; approval numbers were 002365 and 1895499.

### 2.2. Participants and Procedures

Participants were recruited in Florida from continuum care retirement facilities, assisted living facilities, hospitals, independent living facilities, and outpatient medical centers via oral presentations, flyers, and word-of-mouth advertisements. Box 1 outlines the inclusion and exclusion criteria for study participation. Interested participants (or their caregivers) were contacted by phone to determine eligibility. Eligibility was also confirmed through an online survey via Qualtrics [14] or in-person. Once eligibility was confirmed, participants and their primary caregivers were given access to the electronic informed consent form. Interested participants and caregivers without email access were invited to schedule an in-person visit to determine eligibility and, if appropriate, informed consent. All participants were required to schedule an in-person visit to affirm the informed consent and complete the following questionnaires: health history and activity engagement questionnaires, and the Montreal Cognitive Assessment (MoCA). Among older adults with decreased cognition, the primary caregiver completed the survey on behalf of the older adult. The MoCA assessments were administered by trained research assistants at their respective institutions under the guidance of the primary investigator, who conducted the training. The assessments were administered in a private room, in-person at the respective participating Universities’ research laboratories.

A total of 417 individuals were included, comprising 255 females (61%) and 162 males (39%). The average age was 71.98 years (SD ± 8.67). There were 344 (82.5%) individuals who identified as non-Hispanic/Latino, 294 (70.7%) as White, 134 (32.1%) reported having an undergraduate-level education, and 316 (75.8%) self-reported a medium economic status. Out of the 417 participants, 44 had a MoCA score of ≤17, 162 had a MoCA score between 18 and 25, and 211 had a MoCA score between 26 and 30.

Box 1 **Inclusion and Exclusion Criteria.**

Inclusion Criteria

- Age 60 and above.
- Subjects with
  ○ With normal cognitive functions
  ○ Mild cognitive impairment
  ○ Alzheimer’s Disease related dementia.
- Participants who can provide informed consent with English reading and speaking abilities

 

Exclusion Criteria

- History of Brain and Gut related surgeries in the last five years
- History of taking antibiotics in the last 30-days, diarrhea, vomiting, inflammatory bowel disease, food poisoning,
- History of Cancer, except for non-Melanoma skin cancer
- History of neurological disorders like epilepsy, Parkinson’s disease and ALS
- Weight loss more than 10 pounds in 2 weeks.
- Cannot communicate in English.

### 2.3. Study Instruments

The health history questionnaire, adapted from the Florida Department of Elder Affairs, examined sociodemographic information, health behaviors, and medical history [15]. This screening form is standardized, state-endorsed, and widely used in Florida to screen for and prioritize service needs among older adults who seek state-sponsored resources [15,16]. Sociodemographic data were collected to understand the characteristics of the participants. Age was measured as a continuous (years) and a categorical variable. Age categories were designed using 5-year intervals to provide greater analytical precision for studying age-related phenomena [17,18]. Ethnicity was defined as Not Hispanic or Latino, Hispanic or Latino. Race was conceptualized as White, African American, and Asian. Education was defined as High School, Undergraduate, Professional degree/Certificate, Graduate, Medical Doctor (MD), or Doctor of Philosophy (PhD). Economic status was defined as High, Medium, or Low-income status. Health behaviors include smoking and drinking (alcohol) consumption. Smoking frequency was defined by Used in past 30 days (>100 cigarettes), Not used in the past 30 days (>100 cigarettes), Former Smokers, and Never Smokers. Drinking status included Current Drinker, Former, and Never. Medical history questions examined clinical diagnoses and were dichotomized as yes/no. The survey asked about the following ailments: hypertension, diabetes mellitus, non-Melanoma skin cancer, osteoarthritis, stroke, osteoporosis, heart failure, depression, head injury, surgery, substance use, infection, and sound sleep. In addition, we examined the number of chronic diseases as a continuous variable.

Activity engagements were assessed by activity type across various frequencies. Walking and exercise were measured in frequency per week, including Daily, ≤4 times per week, and ≤2 times per week. Domestic chores, driving, leisure activity, using new technology, social activity, cinema theater, gardening, looking after grandchildren, voluntary work, and artistic activities were also measured with weekly frequencies and measured as Often/Always (≥3 times per week) and Never/Rarely (≤2 times per week). The following activities were measured by the annual frequencies of Often/Always (≥3 times in a year) and Never/Rarely (≤2 times in a year), and included exhibitions/conferences/concerts, journeys lasting several days, and reading books. Pet care and managing current accounts were measured as fixed frequencies of Often/Always and Never/Rarely.

We employed the Montreal Cognitive Assessment (MoCA) to screen for cognitive health. The MoCA is a brief, valid, and reliable cognitive screening measure that takes approximately 10 min to complete; it is widely used among older adults to screen for cognitive health [19,20,21,22]. The MoCA consists of 12 tasks that are grouped into cognitive domains of (1) visuospatial/executive functioning, (2) naming, (3) attention, (4) language, (5) abstraction, (6) memory, and (7) orientation [23]. A total score of 30 was possible, encompassing the following categories: 30 to 26—normal cognition, 25 to 18—mild cognitive impairment, and ≤17—moderate to severe cognitive impairment [23].

### 2.4. Statistical Analysis

Our data sample demographic structure and population aged 60 and over in Florida significantly differed from established norms as related to the U.S. Census Bureau [24]. Thus, this study weighted the sample using established population norms. The weighting method employed the “multivariable repeated weighting” (Raking) method, where weights were adjusted sequentially based on each demographic variable. Weighting was accomplished using the SPSS RAKE procedure [25], which simultaneously balanced the distributions of all variables using the GENLOG procedure. The adjusted and weighted sample structure is representative of the reference population (individuals aged 60 and over in Florida) from the U.S. Census Bureau, in terms of age, gender, ethnicity, race, and educational level. Subsequent analyses were done using the weighted estimated sample structure [24].

Descriptive analyses were conducted to describe the sample population by means and frequencies for continuous and categorical variables. The study provided a comparative analysis using One-way Analysis of Variance (ANOVA) for continuous variables such as age, total drinks per week, and number of chronic diseases among different levels of MoCA scores. We used chi-square analyses for categorical variables. Post hoc analyses were performed using the Bonferroni method. All statistical analyses were completed using the Statistical Package for Social Sciences, SPSS (SPSS 29.0, IBM Corp., Chicago, IL, USA), and a two-tailed *p*-value < 0.05 was considered statistically significant [25].

## 3. Results

### 3.1. Demographic Characteristics

Table A1 (in the Appendix A) compares unweighted and weighted sample distributions to target population benchmarks. Table 1 presents the characteristics of study participants categorized by MoCA score. Results suggested that individuals who scored ≤17 points on the MoCA were significantly associated with an older average age of 84.3 years (*p* = 0.001). Significant differences were observed in gender, race, ethnicity, educational level, and economic status by MoCA score categories. Post hoc analyses revealed significant age-related differences among the three MoCA groups. Participants who scored ≤17 points had a significantly older mean age than those in the higher MoCA score groups. Age group analysis revealed that participants under 74 years of age had a higher proportion of higher MoCA scores. In contrast, the group with ≤17 points had a significantly higher proportion of individuals ≥ 85 than those in the higher MoCA score groups. Gender comparisons revealed that the proportion of females was significantly higher in the ≤17 group compared to the 26–30 and 18–25 points groups, while males were more represented in the higher MoCA groups. For ethnicity, Hispanic or Latino individuals were significantly more likely to be in the ≤17 points group than the non-Hispanic or Latino group, particularly in contrast to the two higher MoCA score groups. The 18–25 points group had the highest proportion in all racial categories, except for those indicating ‘other races’. Participants with less than a high school education were significantly higher in the ≤17 points group and had a significantly lower proportion than participants in both the college-educated and undergraduate and higher–educated groups. Lastly, economic status revealed a significant difference between the low-income and medium-income groups, particularly between the ≤17 and 18–25 MoCA points groups.

### 3.2. Health Behavior

Across the three groups, statistically significant differences were found in health behaviors, see Table 2. Older adults with ≤17 points had significantly lower engagement in smoking and drinking. Post hoc analyses revealed that the ≤17 points group had the highest proportion of Never Smokers and drinkers compared to the 26–30 and 18–25 points groups. Statistical significance was also found in walking and exercise behavior across the cognitive health levels (*p* > 0.001). Daily walkers were significantly more frequent in the ≤17 points group, and daily exercise was more frequent in the 18–25 points group, with significant differences.

### 3.3. Activity Engagement

The frequency distribution of activities was statistically significant, except for the frequency of looking after grandchildren (*p* > 0.05), as noted in Table 3. Results suggested that older adults who scored ≤17 points on the MoCA had significantly lower engagement in these activities. Post hoc analyses revealed that those who reported never or rarely engaging were more common in the ≤17 point group, with significant differences from the 26–30 and 18–25 groups.

### 3.4. Clinical Characteristics

The clinical characteristics of older adults differed across cognitive levels. Older adults who scored ≤17 MoCA points reported higher rates of hypertension, osteoarthritis, depression, surgery, and infection compared with individuals who scored higher on the MoCA assessment; see Table 4. Post hoc analyses revealed that the prevalence of hypertension, osteoarthritis, stroke, and depression increased among older adults as MoCA scores declined. In addition, statistically significant differences were observed in the number of chronic diseases across cognitive levels (*p* < 0.001). The average number of chronic diseases was 3.40 (SD ± 2.13) and was highest among older adults who scored ≤17 points on the MoCA, revealing a trend toward lower cognitive scores (Table 4).

## 4. Discussion

We aimed to investigate participants’ engagement in activities at various cognitive health levels while also considering demographic characteristics and clinical history. The study examined differences in demographic, engagement, and clinical characteristics among older adults based on their cognitive performance, as assessed by MoCA scores. Individuals with the lowest scores tended to be older, female, Hispanic or Latino and had lower educational attainment. The findings support our hypothesis that meaningful engagement declines as performance on the MoCA decreases. Older adults with decreased cognitive function participated less in domestic, social, and recreational activities but were more likely to engage in walking and exercise. Health-related behaviors also varied by cognitive level. Older adults with lower scores reported decreased engagement in smoking or drinking. Clinically, individuals with lower cognitive scores reported more health issues, including chronic conditions such as hypertension, osteoarthritis, depression, and infection. The number of chronic illnesses was highest among those with the lowest cognitive scores, suggesting a link between poorer cognitive health and greater medical burden.

### 4.1. Demographic Characteristics

Our results revealed a statistically significant difference between age and MoCA score, indicating that cognitive performance on the MoCA decreased as age increased. This result was not surprising as the literature well establishes the association between age and cognitive performance; changes in brain structure, function, and cognitive reserve occur in the aging process and may contribute to cognitive health [26,27]. Our findings indicate that more females than males had ≤17 MoCA points. The Global Burden of Disease Study 2019 forecasted that the prevalence of dementia in 2050 will be higher for females than males [1]. Our study adds to the literature by examining demographic factors across various levels of cognitive health rather than cognitive status as a dichotomous (yes/no) variable. The association between gender and cognitive health screening tests has been a topic of controversy in the literature; some studies have suggested the importance of considering gender [26,28], while other studies have indicated it is not associated with cognitive performance [29,30]. Due to the longer life expectancy for females, the dementia prevalence rate is expected to be higher for females than for males; females may live longer with decreased cognition and have a greater probability of reporting on cognitive status [31]. Among racial groups, more White than Black and Asian people were noted to have 18–25 MoCA points; non-Hispanic persons showed higher MoCA scores than Hispanic persons. Other studies suggested that the incidence rate for ADRD is increasing among minority older adults [32,33,34]. Our study found that MoCA scores were higher among individuals with higher educational attainment. Older adults with higher educational attainment may have greater brain reserves to tolerate pathological changes in brain structure; a greater cognitive reserve can delay the clinical manifestation of dementia [31,35]. Moreover, higher levels of education may be associated with a healthier lifestyle; studies suggest that education is associated with a lower risk of dementia due to socioeconomic and lifestyle differences [36,37].

### 4.2. Health Behaviors

Our study found that some health behaviors were associated with MoCA levels. Our results on drinking (alcohol consumption) align with previous literature that suggested that, as cognition declines, drinking behavior declines [38]. Among individuals with 18–25 MoCA scores, those who identified as Heavy, Moderate, or Low drinkers were more prevalent overall, while individuals with MoCA scores ≤17 were more likely to be Never Drinkers. Notably, Heavy Drinkers tended to score between 26 and 30 on the MoCA. Our results support professional recommendations that older adults with decreased cognition should not drink alcohol, as this can exacerbate symptoms [39,40]. Prior literature suggested a non-linear risk of alcohol and decreased cognition, while another study suggested that there is a linear association [41,42]. Our study suggested that people with a ≤17 MoCA were Never or Former Smokers. As a percentage, older adults with MoCA >26 were active smokers; however, most of the study participants were Never Smokers. These results are inconclusive; however, prior research found an increased risk of dementia among smokers [43,44]. One study suggested an association between current smoking and decreased cognition among White older adults but not Non-Hispanic Black older adults [45]. The authors also suggested that current smoking may exacerbate late-onset dementia but no significant association between smoking status and cognition [45]. Compared to Never Smokers, Current Smokers had an increased risk of developing ADRD [43]. One study noted that smoking was associated with vascular dementia rather than disease ailments such as Alzheimer’s Disease and Lewy Body Dementia [46]. This indicated a possible association between smoking and types of dementia; however, more research is needed in this area. Other research suggested the synergistic effect of smoking and decreased education on dementia risk [47]. Zhang et al.’s (2024) study suggested that a one-level increase in education was associated with a 7.89% reduction in the probability of smoking initiation [48]. Zhang et al.’s (2024) study suggested that a one-level increase in education was associated with a 7.89% reduction in the probability of smoking initiation [48]. The overall educational status of the sample may contribute to these results, as higher education was associated with a decrease in smoking behavior.

### 4.3. Activity Engagement

Our study found that participants with higher MoCA scores were more frequently engaged in managing accounts, pet care, reading, travel, artistic activities, volunteer work, gardening, social activities, using new technology, leisure activities, and driving. Similarly, previous research demonstrated that individuals with decreased cognition are less likely to participate in complex activities [49,50]. For example, individuals with mild ADRD were less engaged in household chores compared to older adults without dementia due to the physical and cognitive challenges associated with completing the activity independently [49,50]. The increased dependency on a caregiver for support could decrease engagement in complex tasks. However, Huizenga and colleagues noted that older adults, with the appropriate level of assistance, could benefit from performing domestic chores [12]. The authors found that individuals with dementia who continued to participate in household chores could maintain meaningful daily routines; domestic chores provided an avenue for engaging in routine activities and avoiding a sedentary lifestyle. Our study results align with medical recommendations that older adults with decreased cognition should cease driving due to impaired physical and cognitive abilities that may compromise safety [51,52]. Engagement with technology was lower among people with lower MoCA scores. Prior research has found a notable difference between older adults with and without cognitive impairment in terms of technology use [48,49]. Given the complexities of current technology, it is not unreasonable that older adults with lower MoCA scores (≤17) were less likely to use technology [53]. While the literature noted many benefits to using technology, barriers to its use thwarted efforts [49]. Many studies cited user interface, navigational challenges, cost, and computer literacy as barriers to the use of technology [54,55]. Due to cognitive bias, cognitively sensitive technologies were found to better assist older adults with engagement. For example, Stay-Tuned Radio is a communication system designed as a voice messaging application [56]. Pictures of the person sending the voice recording appear with the recording so that the older adult with dementia can select the picture to hear the message. The interaction was relatively simple and reminiscent of a radio interface [57]. VITA, a music pillow, is designed for people with dementia to support engagement with sound and music; it provides a soft touch sensor integrated into the pillow so that older adults with dementia can touch it to activate sound files [58]. Additionally, reminiscence technologies provide a way of evoking familiarity for older adults with ADRD.

Our results showed statistically significant differences between the frequency of physical activity, such as walking and exercise, and cognitive status. Many older adults in the study engaged in physical activities such as walking, and relatively few participants participated in exercise. Many older adults with ≤17 MoCA points walked daily, whereas only 42% of older adults with 26–30 MoCA points walked daily. Research suggested that caregivers and older adults with decreased cognition preferred regular walking due to its adaptability [59,60]. Engaging in more complex exercises varied depending on the caregiver’s capacity and the intensity of cognitive decline and physical mobility, which led to safety concerns. Due to the nature of this study, individuals without caregivers were excluded, and results may differ if individuals with decreased cognition and without caregiver assistance were included.

Similar to our findings, prior research has also discovered a significant association between pet ownership and cognitive status. Most older adults with higher MoCA scores engaged in pet care, and engagement in pet care declined with decreased MoCA scores. In a study examining the association between pet status and patients with mild cognitive impairment, pet ownership did not reduce the risk of dementia, as prominent factors influencing dementia risk were biological, psychological, and social [61]. However, older adults who owned a pet, particularly a dog, were more likely to walk for 3 h per week than those without a dog, indicating that pet care could promote physical activity [62]. Dog walking had a positive psychosocial impact on depressive symptoms and increased social support from pet companionship that may influence dementia severity over time [61].

Overall, older adults with dementia reported a decrease in activity, suggesting the need to improve the activity level, particularly social and emotional engagement of people with decreased cognition. As cognitive function declines due to aging or neurodegenerative conditions, integrating physical and mental activities becomes increasingly important for preserving cognitive health and overall well-being. Mental stimulation through leisure activities such as reading, problem solving, and social engagement has significantly reduced the risk of cognitive impairment, supporting neuroplasticity and cognitive reserve. Concurrently, physical activity has demonstrated a modest but statistically significant association with improved global cognition and reduced incidence of cognitive decline. A meta-analysis of 104 studies involving over 340,000 participants found that physical activity was associated with better late-life cognition, particularly in episodic memory and verbal fluency domains [63]. Similarly, a systematic review of longitudinal studies reported that individuals with higher levels of physical activity had a 35% lower risk of cognitive decline, and a 14% lower risk of dementia compared to less active peers [64]. These findings underscore the importance of multidimensional interventions that combine physical, cognitive, and social engagement to support cognitive health, emotional resilience, and functional independence in aging populations.

Engaging older adults with dementia in meaningful activities is a critical component of person-centered care that supports emotional well-being, reduces behavioral symptoms, and enhances quality of life. Meaningful activity is defined not merely by participation but by its alignment with the individual’s personal history, preferences, and cognitive capacity. Wenborn (2017) emphasized that activities should be embedded into daily routines to foster a culture of engagement and inclusion [65]. This approach aligns with Kitwood’s (1997) framework of psychological needs—comfort, attachment, inclusion, identity, and occupation—emphasizing the importance of meaningful occupation in maintaining personhood in dementia care [66]. In parallel, digital literacy programs have emerged as practical tools for engagement, particularly when instructional strategies are tailored to older adults. Ahmad et al. (2022) identified six evidence-based instructional strategies, collaborative learning, informal settings, use of teaching aids, relevance to personal interests, thoughtful lesson design, and feedback mechanisms, that enhance learning and participation among older adults [67]. These strategies are especially relevant when digital tools are used for reminiscence therapy, communication, or leisure. Xu et al. (2025) further supported intergenerational learning, peer tutoring, and dialogic learning to address the cognitive, social, and emotional needs of older adults with dementia, noting that personalized pacing and long-term support are essential for sustained engagement [68]. These findings collectively highlight that meaningful activity provision must be holistic, culturally sensitive, and adaptable to the evolving needs of individuals with dementia, ensuring that care environments promote dignity, autonomy, and connection.

### 4.4. Clinical Characteristics

Our findings indicated that participants with MoCA scores varied according to their clinical characteristics. Our results show that people with decreased MoCA scores with non-melanoma skin cancer reported scores 18–25; however, no conclusive association can be made from these data, as more individuals with cognitive decline reported no non-melanoma cancer, and cancer was narrowly defined in this study, and a sizeable proportion remains in the Unknown category. However, previous research reported that older adults with advanced cancer (more broadly defined) were at a higher risk for treatment-toxic effects. Among patients with cancer who were ≥65 years old, cognitive impairment was reported in 36% of patients [68]. A web-based survey revealed that 75% of cancer patients reported cognitive complaints related to cancer treatments [69]. Research suggests that physical activity may improve both mental and physical well-being; however, it does not provide specific activity recommendations due to variations in cancer and treatment effects [70,71].

Our study results did not suggest a link between cognition and diabetes among older adults. Dove et al. (2021) suggested that diabetes was linked to dementia, and poorly controlled diabetes can exacerbate the progression of dementia, particularly among people with low socioeconomic status and low educational attainment [72]. Other literature suggested that the early age of onset of diabetes has a significant role in dementia diagnosis, while other studies suggested that well-controlled glycemia was protective against dementia [73,74]. However, more research is needed to conclusively determine associations. Our study found that older adults who reported diagnoses of hypertension, stroke, head injury, osteoarthritis, and depression tended to have lower MoCA scores. Growing evidence has highlighted the age-dependent hypertension-cognition relationship [66], suggesting that mitigating hypertension at midlife may have significant effects on dementia prevalence [75]. However, the data did not support a direct association between stroke and dementia, as a greater proportion of participants without a history of stroke had decreased MoCA scores. Stroke itself may not be an independent risk factor for dementia; evidence suggests that underlying vascular risk contributes to both stroke and cognitive decline [74]. Consequently, individuals who experience a stroke may be at increased risk for accelerated cognitive deterioration due to shared vascular pathology [75]. Rost et al. (2022) study found that stroke may increase the risk of ADRD due to microbleeds and lesions that may compromise brain health [76]. Our study suggests that heart failure is not associated with decreased cognition. The literature is inconclusive regarding this association. Ren and colleagues (2023) suggested that there is an association between heart failure and dementia [77]. However, a meta-analysis and systematic review suggested mixed results on the association between heart failure [78]. Currently, most heart failure guidelines do not include cognitive screenings, but perhaps guidance and research should consider the severity of heart failure and dementia subtypes [78,79].

As supported by prior literature, the number of chronic diseases increased with cognitive decline [80]. Maintaining clinical health is critical in mitigating cognitive decline, particularly in aging populations. Access to consistent and high-quality primary care was associated with a reduced risk of cognitive impairment, mainly through the management of modifiable risk factors such as hypertension, diabetes, and dyslipidemia [81]. Individuals residing in areas with primary care shortages or high emergency department utilization were found to have significantly higher odds of cognitive impairment, highlighting the role of healthcare infrastructure in cognitive health outcomes. Furthermore, early identification of cognitive decline through clinical assessments can inform care planning and mitigate adverse medical outcomes. These findings underscore the preventive role of clinical health services in identifying and managing cognitive deterioration, thereby enhancing patient outcomes and optimizing healthcare resource utilization.

### 4.5. Limitations

The study results should be considered in light of the limitations. The self-reported questionnaire could introduce recall bias for participants or caregivers. Such misclassifications would bias our results, thus reducing the ability to detect statistical significance between variables and cognitive levels. Selection bias due to purposive sampling and the underrepresentation of minority groups in participation may influence the study results. The dichotomous nature of the questionnaire may limit the complete understanding of clinical characteristics. For example, reporting cancer as yes or no without specifying the type or intensity (stage) of cancer may be limiting. The initial sample size was relatively small, but we attempted to overcome this limitation by weighting the sample, a commonly accepted practice in research [82]. While this study examines activity engagement across cognitive stages, future research should investigate the influence of caregivers on activity engagement, as caregiver burden may impact their ability to support engagement. For example, while individuals with lower MoCA scores demonstrated greater participation in physical activities such as walking, they were notably less involved in social and caregiving roles. Additionally, future research may investigate the role of caregivers in various engagement activities. Such research could inform targeted interventions designed to support caregivers in community-based settings. Future studies should also control demographic and social factors that may bias results. Subsequent analysis should incorporate clinically confirmed medical diagnoses with greater specificity to better understand their relationship to cognitive performance and meaningful engagement. In particular, conditions such as heart failure, stroke, various cancer types, and the onset and progression of diabetes should be examined in more detail. Differentiating these diagnoses by subtype, severity, and timing may reveal distinct cognitive and behavioral patterns, allowing for more nuanced analysis and targeted interventions.

## 5. Conclusions

Our study results suggest that activity engagement differs at various cognitive health stages. People with the highest MoCA scores were engaged in meaningful activities; engagement in activities declined alongside cognitive status. These findings underscore the importance of promoting activities that may promote quality of life in older adults. Engagement should be meaningful and promote quality of life with the appropriate level of assistance.

## Figures and Tables

**Table 1:** Summary of Participant Characteristics.

	All	MoCA Score				
		26–30 Points	18–25 Points	≤17 Points		
	(n = 417)	(n = 211)	(n = 162)	(n = 44)	*p*-Value	Post Hoc
Age (mean, SD)	71.98 ± 8.67	70.61 ± 6.53	70.39 ± 8.48	84.30 ± 8.60	<0.001 ^b^	[≤17 points] > [26–30 points], [≤17 points] > [18–25 points]
60 to 64 years	102 (24.5)	46 (21.8)	56 (34.6)	0 (0)	<0.001 ^b^	[26–30 points] > [≤17 points], [18–25 points] > [≤17 points]
65 to 69 years	91 (21.8)	54 (25.6)	36 (22.2)	1 (2.3)		[26–30 points] > [≤17 points], [18–25 points] > [≤17 points]
70 to 74 years	80 (19.2)	61 (28.9)	12 (7.4)	7 (15.9)		[26–30 points] > [18–25 points], [26–30 points] > [≤17 points], [18–25 points] > [≤17 points]
75 to 79 years	67 (16.1)	28 (13.3)	35 (21.6)	4 (9.1)		-
80 to 84 years	42 (10.1)	18 (8.5)	17 (10.5)	7 (15.9)		-
≥85 years	35 (8.4)	4 (1.9)	6 (3.7)	25 (56.8)		[≤17 points] > [26–30 points], [≤17 points] > [18–25 points]
Gender, n (%)					<0.001 ^b^	
Male	192 (46.0)	88 (41.7)	98 (60.5)	6 (13.6)		[26–30 points] > [≤17 points], [18–25 points] > [≤17 points], [18–25 points] > [26–30 points]
Female	225 (54.0)	123 (58.3)	64 (39.5)	38 (86.4)		[26–30 points] > [18–25 points], [≤17 points] > [18–25 points], [≤17 points] > [26–30 points]
Ethnicity, n (%)					<0.001 ^b^	
Not Hispanic or Latino	344 (82.5)	184 (87.6)	143 (88.3)	17 (37.8)		[26–30 points] > [≤17 points], [18–25 points] > [≤17 points]
Hispanic or Latino	73 (17.5)	26 (12.4)	19 (11.7)	28 (62.2)		[26–30 points] > [≤17 points], [18–25 points] > [≤17 points]
Race, n (%)					<0.001 ^b^	
White	294 (70.7)	131 (62.4)	130 (80.2)	33 (75.0)		[18–25 points] > [26–30 points], [18–25 points] > [≤17 points]
African American	41 (9.9)	11 (5.2)	26 (16.0)	4 (9.1)		[18–25 points] > [26–30 points], [18–25 points] > [≤17 points]
Asian	10 (2.4)	3 (1.4)	6 (3.7)	1 (2.3)		-
Others	71 (17.1)	65 (31.0)	0 (0)	6 (13.6)		[26–30 points] > [18–25 points], [≤17 points] > [18–25 points]
Education, n (%)					<0.001 ^b^	
Less than high school	44 (10.6)	12 (5.7)	11 (6.8)	21 (47.7)		[≤17 points] > [18–25 points], [≤17 points] > [26–30 points]
High school	119 (28.5)	45 (21.3)	62 (38.3)	12 (27.3)		[18–25 points] > [26–30 points]
College	120 (28.8)	66 (31.3)	50 (30.9)	4 (9.1)		[26–30 points] > [≤17 points], [18–25 points] > [≤17 points]
Undergraduate and higher	134 (32.1)	88 (41.7)	39 (24.1)	7 (15.9)		[26–30 points] > [18–25 points], [26–30 points] > [≤17 points]
Economic status, n (%)					0.026 ^a^	
High	65 (15.6)	29 (13.7)	29 (17.9)	7 (15.9)		-
Medium	316 (75.8)	169 (80.1)	111 (68.5)	36 (81.8)		[26–30 points] > [18–25 points], [≤17 points] > [18–25 points]
Low	36 (8.6)	13 (6.2)	22 (13.6)	1 (2.3)		[18–25 points] > [≤17 points], [18–25 points] > [26–30 points]

^a^ Statistical significance *p*-value < 0.05. ^b^ Statistical significance *p*-value < 0.001. Abbreviations: mean = n, standard deviation = SD.

**Table 2:** Summary of Health Behaviors.

	All	26–30 Points	18–25 Points	≤17 Points		
	(n = 417)	(n = 211)	(n = 162)	(n = 44)	*p*-Value	Post Hoc
Smoking status, n (%) ^c^					<0.001 ^b^	
Used in past 30 days	21 (5.1)	8 (3.8)	14 (8.6)	0 (0)		-
Not used in past 30 days	21 (5.1)	2 (0.9)	18 (11.1)	0 (0)		[26–30 points] > [≤17 points]
						[18–25 points] > [≤17 points]
						[18–25 points] > [26–30 points]
Former	117 (27.9)	55 (26.1)	58 (35.8)	4 (8.9)		[26–30 points] > [≤17 points]
						[18–25 points] > [≤17 points]
Never	258 (61.9)	146 (69.2)	72 (44.4)	40 (91.1)		[26–30 points] > [18–25 points]
						[≤17 points] > [18–25 points]
						[≤17 points] > [26–30 points]
Drinking status, n (%)					<0.001 ^b^	
Current drinker	158 (37.9)	100 (47.4)	57 (35.2)	1 (2.3)		[26–30 points] > [≤17 points]
						[18–25 points] > [≤17 points]
Former	215 (51.6)	102 (48.3)	98 (60.5)	15 (34.1)		[18–25 points] > [≤17 points]
Never	44 (10.6)	9 (4.3)	7 (4.3)	28 (63.6)		[≤17 points] > [18–25 points]
						[≤17 points] > [26–30 points]
Total drinks/week (mean, SD)	3.07±6.43	3.64±3.68	3.15±9.27	0.05±0.48	0.003 ^a^	[26–30 points] > [≤17 points]
						[18–25 points] > [≤17 points]
Heavy drinkers	158 (37.9)	100 (47.4)	57 (35.2)	1 (2.3)		[26–30 points] > [≤17 points]
						[18–25 points] > [≤17 points]
Low to moderate drinkers	215 (51.6)	102 (48.3)	98 (60.5)	15 (34.1)	<0.001 ^b^	-
Never drinking	44 (10.6)	9 (4.3)	7 (4.3)	28 (63.3)		[≤17 points] > [18–25 points]
						[≤17 points] > [26–30 points]
Walking n (%)					<0.001 ^b^	
Daily	209 (50.0)	89 (42.0)	81 (50.0)	39 (86.7)		[≤17 points] > [18–25 points]
						[≤17 points] > [26–30 points]
≤4 times/week	100 (24.0)	77 (36.3)	20 (12.3)	3 (6.7)		[26–30 points] > [18–25 points]
						[26–30 points] > [≤17 points]
≤2 times/week	82 (19.7)	36 (17.1)	44 (27.2)	2 (4.4)		[18–25 points] > [≤17 points]
Never	15 (3.7)	1 (0.5)	14 (9.3)	0 (0)		[18–25 points] > [26–30 points]
Unknown	11 (2.6)	9 (4.2)	2 (1.2)	0 (0)		-
Exercise n (%)					<0.001 ^b^	
Daily	64 (15.4)	23 (10.9)	38 (23.5)	3 (6.8)		[18–25 points] > [26–30 points]
						[18–25 points] > [≤17 points]
≤4 times/week	100 (24.0)	44 (20.9)	44 (27.2)	12 (27.3)		–
≤2 times/week	143 (34.3)	105 (49.8)	38 (23.5)	0 (0)		[26–30 points] > [18–25 points]
						[26–30 points] > [≤17 points]
						[18–25 points] > [≤17 points]
Never	46 (10.9)	19 (9.0)	23 (14.2)	3 (6.8)		–
Unknown	64 (15.4)	20 (9.4)	19 (11.7)	26 (59.1)		[≤17 points] > [18–25 points]
						[≤17 points] > [26–30 points]

^a^ Statistical significance *p*-value < 0.05, ^b^ Statistical significance *p*-value < 0.001; ^c^ Used in past 30 days (>100 cigarettes): Smoked > 100 cigarettes in my lifetime but did not smoke in the last month; Not used in past 30 days (>100 cigarettes): Smoked > 100 cigarettes in my lifetime but did not smoke in the last month. Former: Smoked < 100 cigarettes in their lifetime, Never in 100 days, and not smoked in the past five years.

**Table 3:** Summary of Activity Engagements.

	All	26–30 Points	18–25 Points	≤17 Points		
	(n = 417)	(n = 211)	(n = 162)	(n = 44)	*p*-Value	Post Hoc
Activities with weekly frequency, n (%)						
Reading newspapers and magazines					<0.001 ^b^	
Often/always (≥3 times per week)	263 (63.1)	151 (71.9)	102 (63.0)	10 (22.2)		[26–30 points] > [≤17 points]
						[18–25 points] > [≤17 points]
Never/rarely (≤2 times per week)	152 (36.5)	58 (27.6)	60 (37.0)	34 (75.6)		[26–30 points] > [≤17 points]
						[18–25 points] > [≤17 points]
Unknown	2 (0.5)	1 (0.5)	0 (0)	1 (2.2)		-
Domestic chores					<0.001 ^b^	
Often/always (≥3 times per week)	357 (85.6)	196 (92.9)	151 (93.2)	10 (22.7)		[26–30 points] > [≤17 points]
						[18–25 points] > [≤17 points]
Never/rarely (≤2 times per week)	58 (14.0)	14 (6.6)	11 (6.8)	33 (75.0)		[26–30 points] > [≤17 points]
						[18–25 points] > [≤17 points]
Unknown	2 (0.4)	1 (0.5)	0 (0)	1 (2.3)		-
Driving					<0.001 ^b^	
Often/always (≥3 times per week)	346 (82.9)	199 (94.3)	143 (86.3)	4 (9.1)		[26–30 points] > [≤17 points]
						[18–25 points] > [≤17 points]
Never/rarely (≤2 times per week)	65 (15.7)	11 (5.2)	14 (8.6)	40 (90.9)		[≤17 points] > [18–25 points]
						[≤17 points] > [26–30 points]
Unknown	6 (1.4)	1 (0.5)	5 (3.1)	0 (0)		-
Leisure activity					<0.001 ^b^	
Often/always (≥3 times per week)	239 (57.3)	146 (69.2)	83 (51.2)	10 (22.7)		[26–30 points] > [18–25 points],
						[26–30 points] > [≤17 points]
						[18–25 points] > [≤17 points]
Never/rarely (≤2 times per week)	176 (42.2)	63 (29.9)	79 (48.8)	34 (77.3)		[≤17 points] > [18–25 points]
						[≤17 points] > [26–30 points]
						[18–25 points] > [26–30 points]
Unknown	2 (0.5)	2 (0.9)	0 (0)	0 (0)		-
Using new technology					<0.001 ^b^	
Often/always (≥3 times per week)	361 (86.6)	196 (92.9)	156 (96.3)	9 (20.5)		[26–30 points] > [≤17 points]
						[18–25 points] > [≤17 points]
Never/rarely (≤2 times per week)	53 (12.7)	14 (6.6)	5 (3.1)	34 (77.3)		[≤17 points] > [18–25 points]
						[≤17 points] > [26–30 points]
Unknown	3 (0.7)	1 (0.5)	1 (0.6)	1 (2.3)		**-**
Social activity					<0.001 ^b^	
Often/always (≥3 times per week)	178 (42.7)	129 (61.1)	48 (29.6)	1 (2.3)		[26–30 points] > [18–25 points],
						[26–30 points] > [≤17 points]
						[18–25 points] > [≤17 points]
Never/rarely (≤2 times per week)	231 (55.4)	79 (37.4)	109 (67.3)	43 (97.7)		[≤17 points] > [18–25 points]
						[≤17 points] > [26–30 points]
						[18–25 points] > [26–30 points]
Unknown	8 (1.9)	3 (1.4)	5 (3.1)	0 (0)		-
Cinema theater					<0.001 ^b^	
Often/always (≥3 times per week)	78 (18.6)	22 (10.4)	55 (34.0)	1 (2.3)		[18–25 points] > [≤17 points]
						[18–25 points] > [≤17 points]
Never/rarely (≤2 times per week)	321 (77.0)	187 (88.6)	92 (56.8)	42 (95.4)		[26–30 points] > [18–25 points],
						[≤17 points] > [18–25 points]
Unknown	18 (4.3)	2 (0.9)	15 (9.2)	1 (2.3)		[18–25 points] > [26–30 points]
Gardening					<0.001 ^b^	
Often/always (≥3 times per week)	150 (35.9)	78 (37.1)	68 (42.0)	4 (8.9)		[26–30 points] > [≤17 points]
						[18–25 points] > [≤17 points]
Never/rarely (≤2 times per week)	250 (60.1)	131 (62.4)	79 (48.8)	40 (88.9)		[≤17 points] > [18–25 points]
						[≤17 points] > [26–30 points
						[26–30 points] > [18–25 points]
Unknown	17 (4.0)	1 (0.5)	15 (9.2)	1 (2.2)		[18–25 points] > [26–30 points]
Looking after grandchildren					0.099	
Often/always (≥3 times per week)	58 (14.0)	37 (17.5)	20 (12.3)	1 (2.3)		[26–30 points] > [≤17 points]
Never/rarely (≤2 times per week)	349 (83.7)	170 (80.6)	137 (84.6)	42 (95.5)		[≤17 points] > [26–30 points]
Unknown	10 (2.3)	4 (1.9)	5 (8.1)	1 (2.3)		-
Voluntary work					<0.001 ^b^	
Often/always (≥3 times per week)	133 (31.9)	90 (42.7)	42 (25.9)	1 (2.3)		[26–30 points] > [18–25 points]
						[26–30 points] > [≤17 points]
						[18–25 points] > [≤17 points]
Never/rarely (≤2 times per week)	273 (65.5)	116 (55.0)	115 (71.0)	42 (95.5)		[≤17 points] > [18–25 points]
						[≤17 points] > [26–30 points]
						[18–25 points] > [26–30 points]
Unknown	11 (2.6)	5 (2.4)	5 (3.1)	1 (2.3)		-
Artistic activities					0.003 ^a^	
Often/always (≥3 times per week)	98 (23.5)	61 (28.9)	34 (21.0)	3 (6.8)		[26–30 points] > [≤17 points]
Never/rarely (≤2 times per week)	311 (74.7)	149 (70.6)	122 (75.3)	40 (90.9)		[≤17 points] > [26–30 points]
Unknown	8 (1.9)	1 (0.5)	6 (3.7)	1 (2.3)		-
Activities with annual frequency, n (%)						
Exhibitions, Concerts, Conferences					<0.001 ^b^	
Often/always (≥3 times in a year)	152 (36.4)	106 (50.2)	43 (26.5)	3 (6.8)		[26–30 points] > [18–25 points]
						[26–30 points] > [≤17 points]
						[18–25 points] > [≤17 points]
Never/rarely (≤2 times in a year)	259 (62.2)	102 (48.3)	118 (72.8)	39 (88.6)		[18–25 points] > [26–30 points]
						[≤17 points] > [26–30 points]
Unknown	6 (1.4)	3 (1.4)	1 (0.6)	2 (4.5)		-
Journeys last several days					<0.001 ^b^	
Often/always (≥3 times in a year)	184 (44.2)	135 (64.0)	45 (27.8)	4 (9.1)		[26–30 points] > [18–25 points]
						[26–30 points] > [≤17 points]
						[18–25 points] > [≤17 points]
Never/rarely (≤2 times in a year)	229 (54.8)	73 (34.6)	117 (72.2)	39 (88.6)		[18–25 points] > [26–30 points]
						[≤17 points] > [26–30 points]
Unknown	4 (1.0)	3 (1.4)	0 (0)	1 (2.3)		-
Reading books					0.007 ^a^	
Often/always (≥3 times in a year)	197 (47.2)	101 (47.9)	86 (53.1)	10 (22.7)		[26–30 points] > [≤17 points]
						[18–25 points] > [≤17 points]
Never/rarely (≤2 times in a year)	219 (52.5)	109 (51.6)	76 (46.9)	34 (77.3)		[≤17 points] > [26–30 points]
						[≤17 points] > [18–25 points]
Unknown	1 (0.3)	1 (0.5)	0 (0)	0 (0)		-
Activities with fix frequency, n (%)						
Pet care					<0.001 ^b^	
Often/Always	223 (53.5)	132 (62.6)	83 (50.2)	8 (18.2)		[26–30 points] > [≤17 points]
						[18–25 points] > [≤17 points]
Never/rarely	188 (45.1)	77 (36.5)	75 (46.3)	36 (81.8)		[≤17 points] > [26–30 points]
						[≤17 points] > [18–25 points]
Unknown	6 (1.4)	2 (0.9)	4 (2.5)	0 (0)		-
Managing current account					<0.001 ^b^	
Often/Always	255 (61.2)	147 (69.7)	101 (62.3)	7 (15.9)		[26–30 points] > [≤17 points]
						[18–25 points] > [≤17 points]
Never/rarely	94 (22.5)	27 (12.8)	35 (21.6)	32 (72.7)		[≤17 points] > [26–30 points]
						[≤17 points] > [18–25 points]
Unknown	68 (16.3)	37 (17.5)	26 (16.1)	5 (11.4)		-

^a^ Statistical significance *p*-value < 0.05, ^b^ Statistical significance *p*-value < 0.001.

**Table 4:** Clinical Characteristics by Cognitive Group (n = 417).

	All	MoCA				
	(n = 417)	26–30 Points	18–25 Points	≤17 Points	*p*-Value	Post Hoc
		(n = 211)	(n = 162)	(n = 44)		
Chronic diseases (mean, SD)	3.40 ± 2.13	2.92 ± 1.55	3.70 ± 2.45	4.55 ± 2.62	<0.001 ^b^	[≤17 points] > [26–30 points],
						[≤17 points] > [18–25 points],
						[18–25 points] > [26–30 points]
Hypertension, n (%)					<0.001 ^b^	
Yes	139 (33.3)	41 (19.4)	69 (42.6)	29 (65.9)		[≤17 points] > [26–30 points]
						[≤17 points] > [18–25 points],
						[18–25 points] > [26–30 points]
No	255 (61.2)	159 (75.4)	81 (50.0)	15 (34.1)		[26–30points]> [18–25 points]
						[26–30 points] > [≤17 points]
Unknown	23 (5.5)	11 (5.2)	12 (7.4)	0 (0)		-
Diabetes mellitus, n (%)					0.001 ^b^	
Yes	46 (11.1)	13 (6.2)	30 (18.5)	4 (9.1)		[18–25 points] > [26–30 points]
No	352 (84.6)	191 (90.5)	121 (74.7)	40 (90.9)		[26–30 points] > [18–25 points]
Unknown	18 (4.3)	7 (3.3)	11 (6.8)	0 (0)		-
Non-Melanoma Cancer, n (%)					0.025 ^a^	
Yes	89 (21.3)	43 (20.4)	40 (24.7)	6 (13.6)		-
No	304 (72.8)	161 (76.3)	110 (67.9)	33 (75.0)		-
Unknown	24 (5.9)	7 (3.3)	12 (7.4)	5 (11.4)		[≤17 points] > [26–30 points]
Osteoarthritis, n (%)					0.001 ^b^	
Yes	137 (32.9)	53 (25.1)	60 (37.0)	24 (54.5)		[18–25points]> [26–30 points]
						[≤17 points] > [26–30 points]
No	255 (61.2)	146 (69.2)	89 (54.9)	20 (45.5)		[26–30points]> [18–25 points]
						[26–30 points] > [≤17 points]
Unknown	25 (6.0)	12 (5.7)	13 (8.0)	0 (0)		-
Stroke, n (%)					<0.001 ^b^	
Yes	27 (6.5)	2 (0.9)	3 (1.9)	22 (48.9)		[≤17 points] > [26–30 points]
						[≤17 points] > [18–25 points]
No	363 (87.1)	195 (92.4)	145 (90.1)	23 (51.1)		[26–30 points] > [≤17 points]
						[18–25 points] > [≤17 points]
Unknown	27 (6.5)	14 (6.6)	13 (8.1)	0 (0)		-
Osteoporosis, n (%)					0.017 ^a^	
Yes	77 (18.5)	47 (22.3)	29 (17.9)	1 (2.3)		[26–30 points] > [≤17 points]
						[18–25 points] > [≤17 points]
No	318 (76.3)	156 (73.9)	121 (74.7)	41 (93.2)		[≤17 points] > [26–30 points]
						[≤17 points] > [18–25 points]
Unknown	22 (5.3)	8 (3.8)	12 (7.4)	2 (4.5)		-
Heart failure, n (%)					0.343	
Yes	5 (1.3)	2 (0.9)	3 (1.9)	0 (0)		-
No	385 (92.3)	195 (92.4)	146 (90.1)	44 (100)		-
Unknown	27 (6.4)	14 (6.6)	13 (8.0)	0 (0)		-
HIV infection, n (%)					0.073	
Yes	3 (0.7)	0 (0)	3 (1.9)	0 (0)		-
No	387 (92.8)	197 (93.4)	146 (90.1)	44 (100)		-
Unknown	27 (6.5)	14 (6.6)	13 (8.0)	0 (0)		-
Depression, n (%)					<0.001 ^b^	
Yes	96 (23.0)	30 (14.2)	39 (24.1)	27 (61.4)		[≤17 points] > [26–30 points]
						[≤17 points] > [18–25 points]
						[18–25 points] > [26–30 points]
No	300 (71.9)	171 (81.0)	112 (69.1)	17 (38.6)		[26–30 points] > [≤17 points]
						[18–25 points] > [≤17 points]
						[26–30 points] > [18–25 points]
Unknown	21 (5.0)	10 (4.7)	11 (6.8)	0 (0)		-
Head injury, n (%)					<0.001 ^b^	
Yes	85 (20.5)	19 (9.0)	44 (27.2)	22 (50.0)		[≤17 points] > [26–30 points]
						[≤17 points] > [18–25 points]
						[18–25 points] > [26–30 points]
No	304 (72.9)	177 (83.9)	105 (64.8)	22 (50.0)		[26–30points] > [18–25 points],
						[26–30 points] > [≤17 points]
Unknown	28 (6.6)	15 (7.1)	13 (8.0)	0 (0)		-
Surgery, n (%)					0.001 ^b^	
Yes	302 (72.4)	167 (79.1)	110 (67.9)	25 (56.8)		[26–30points] > [18–25 points]
						[26–30 points] > [≤17 points]
No	97 (23.3)	37 (17.5)	41 (25.3)	19 (43.2)		[≤17 points] > [26–30 points]
Unknown	18 (4.3)	7 (3.3)	11 (6.8)	0 (0)		-
Substance use, n (%)					0.022 ^a^	
Yes	10 (2.4)	2 (1.0)	8 (4.9)	0 (0)		-
No	379 (91.0)	194 (92.4)	141 (87.0)	44 (100)		[≤17 points] > [18–25 points]
Unknown	28 (6.6)	14 (6.7)	13 (8.0)	0 (0)		-
Infection, n (%)					0.004 ^a^	
Yes	139 (33.3)	67 (31.9)	50 (30.7)	22 (50.0)		-
No	251 (60.2)	136 (64.8)	95 (58.3)	20 (45.5)		-
Unknown	27 (6.5)	7 (3.3)	18 (11.0)	2 (4.5)		[18–25 points] > [26–30 points]
Sound sleep, n (%)					0.948	
Yes	123 (29.5)	65 (30.8)	35 (21.6)	23 (52.3)		[≤17 points] > [26–30 points]
						[≤17 points] > [18–25 points]
No	262 (62.8)	131 (62.1)	112 (69.1)	19 (43.2)		[26–30 points] > [≤17 points]
						[18–25 points] > [≤17 points]
Unknown	32 (7.7)	15 (7.1)	15 (9.3)	2 (4.5)		-

^a^ Statistical significance *p*-value < 0.05, ^b^ Statistical significance *p*-value < 0.05, Abbreviations: mean = n, standard deviation = SD.

## Data Availability

The data presented in this study are available on request from the corresponding author due to privacy, ethical, and institutional restrictions associated with the use of geocoded health and demographic data.

## Appendix A

**Table A1:** Weighting structure pre- and post-weighted.

	Florida Data Population 60 Years and Over		Pre-Weighted Data		Weighted Data	
	Number	%	Number	%	Number	%
Total	6,515,453	100.0	417	100.0	417	100.0
Age						
60 to 64 years	1,597,671	24.5	55	13.2	102	24.5
65 to 69 years	1,428,538	21.9	100	24.0	91	21.9
70 to 74 years	1,252,984	19.2	31	7.4	80	19.2
75 to 79 years	1,031,541	15.8	164	39.3	66	15.8
80 to 84 years	657,889	10.1	28	6.7	42	10.1
≥85 years	546,830	8.4	38	9.4	35	8.4
Gender, n (%)						
Male	2,997,108	46.0	151	36.2	192	46.0
Female	3,518,345	54.0	266	63.8	225	54.0
Ethnicity, n (%)						
Not Hispanic or Latino	5,368,733	82.4	379	90.9	344	82.4
Hispanic or Latino	1,146,720	17.6	38	9.1	73	17.6
Race, n (%)						
White	4,606,425	70.7	380	91.1	295	70.7
African American	651,545	10.0	18	4.3	42	10.0
Asian	156,371	2.4	15	3.6	10	2.4
Others	1,101,112	16.9	4	1.0	70	16.9
Education, n (%)						
Less than high school	690,638	10.6	3	0.7	44	10.6
High school	1,856,904	28.5	57	13.7	119	28.5
College	1,869,935	28.7	16	3.8	120	28.7
Undergraduate and higher	2,097,976	32.2	341	81.8	134	32.2
